# Hardness Analysis of Foods in a Diet Based on the Mediterranean Diet and Adapted to Chilean Gastronomy

**DOI:** 10.3390/foods13193061

**Published:** 2024-09-26

**Authors:** Franco Marinelli, Camila Venegas, Fanny Pirce, Jennifer del Carmen Silva Celedón, Pablo Navarro, Marcela Jarpa-Parra, Ramón Fuentes

**Affiliations:** 1 Research Centre in Dental Sciences (CICO-UFRO), Dental School, Facultad de Odontología, Universidad de La Frontera, Temuco 4780000, Chile; marinelli.fran@gmail.com (F.M.); camilabelen.venegas@ufrontera.cl (C.V.); pablo.navarro@ufrontera.cl (P.N.); 2 Agroindustry Institute, Universidad de La Frontera, Temuco 4780000, Chile; fanny.pirce@ufrontera.cl (F.P.); jennifer.silva@ufrontera.cl (J.d.C.S.C.); 3 Facultad de Ciencias de la Salud, Universidad Autónoma de Chile, Santiago 7500000, Chile; 4 Agro-Food Research Center and Vegetable Protein Laboratory, Universidad Adventista de Chile, Chillán 3780000, Chile; marcelajarpa@unach.cl; 5 Department of Integral Adults Dentistry, Dental School, Facultad de Odontología, Universidad de La Frontera, Temuco 4780000, Chile

**Keywords:** TPA analysis, hardness, Mediterranean diet

## Abstract

The human diet is a factor for disease prevention and the extension of life expectancy. Loss of teeth can adversely affect chewing capacity, which can lead patients to modify their diet and subsequently result in a poor dietary intake. This work is conducted within the framework of an ongoing research project in the Dentistry School of Universidad de la Frontera aimed at designing a diet for patients with complete removable dental prostheses (CRDP). This study aimed to evaluate the hardness of foods in a diet designed for patients using CRDP, using texture profile analysis (TPA). TPA was used to measure the hardness of 43 foods, categorized into seven groups, dairy, animal protein, fruits, vegetables, cereals and grains, high-lipid foods, and vegetable protein, to understand their impact on masticatory performance in CRDP wearers. TPA consists of two compression cycles where the food sample is compressed until it reaches a pre-established deformation. The first force peak achieved in the first cycle is used as a measure of sample hardness. Significant differences in hardness were identified within each food group, indicating a wide spectrum of textural properties that could influence chewing behavior. These findings suggest that assessing food hardness can help tailor dietary recommendations to improve masticatory efficiency in patients with dental prostheses.

## 1. Introduction

Diet is an important factor for disease prevention and the extension of life expectancy [1]. The loss of teeth is a critical condition that adversely affects chewing capacity, which can lead patients to modify their diet composition and result in poor dietary intake [2]. This can lead to malnutrition, as certain foods are avoided, such as fruits and vegetables [3]. Gil-Montoya et al. [4] conducted a study with 2000 dentate subjects and 860 edentulous subjects. They found a 10% increase in the risk of malnutrition among edentulous patients. Saarela et al. [5] found that edentulous subjects without dentures present a higher prevalence of malnutrition, 32.1%, according to the Mini Nutritional Assessment, whereas edentulous patients with some removable dentures present 26.1%, and dentulous patients with or without removable dentures present 16.6%. Malnutrition is not only a lack of nutrients, but also an unbalanced nutrient intake. Edentulism (or toothlessness) is related not only to a nutrient deficit [6,7,8,9], but has also has been found to be related to obesity [10,11]. Furthermore, a lower consumption of fruits and vegetables is highly related to a low oral health quality of life (OHQoL) [12,13], which becomes a vicious cycle, where a low OHQoL leads to a lower consumption of fruits and vegetables that further decreases OHQoL.

Partial and complete dentures are the recommended treatment to recover chewing function [14,15]. The use of dentures has a positive impact on maintenance of food forms and nutrient intake, enabling denture wearers to chew [16,17,18,19]. Some studies have reported the effects of chewing on logical tests related to arousal [20], attention [21], reaction time [22], and memory [23]. These studies have shown an improvement in the variable under study when subjects were asked to chew gum for a certain period and then repeat the test. Additionally, there is a broad consensus that associates the loss of masticatory function with cognitive decline, due not only to the lack of stimuli but also to malnutrition resulting from this condition [24,25]. Combined with this, diet design and eating recommendations are a complement that benefit the nutrient condition of patients [26] and even have an impact on cognitive function [27]. Based on the aforementioned, the design of a dietary guideline focused on denture wearers is of interest to improve the quality of life for these patients. Traditional Chilean cuisine shares many similarities with Mediterranean culinary traditions. Dishes like charquicán, porotos granados (stewed beans with corn), and cazuela de ave (chicken stew) use a flavor base of onions, oregano, cumin, paprika, and garlic—like the Spanish sofrito. Chilean seafood soups such as caldillo de congrio (conger eel stew) and mariscal also feature Mediterranean ingredients like tomatoes, peppers, and parsley. Common salads include tomatoes, onions, and herbs, dressed with oil, lemon, and salt. Pebre, a traditional Chilean condiment made with finely chopped onions, tomatoes, garlic, parsley, cilantro, and chili, is typically served with vinegar, salt, and oil. Wine consumption is also integral to Chilean food culture [28].

The Mediterranean diet (MedDiet) is a well-known dietary pattern based on a high intake of plant foods (fruit, vegetable, bread, cereals, potatoes, beans, nuts, and seeds) [29]. Several studies have evaluated the effects of the MedDiet in reducing the risk of obesity [30,31] cardiovascular disease [32], cancer [33], Parkinson’s disease [34], and Alzheimer’s disease [35]. In a recent study, Wu et al. [36] analyzed a group of 8290 people, 4159 participants with periodontitis and 4131 without periodontitis, and found a negative relationship between the presence of periodontitis, probing pocket depth (PPD), clinical attachment level (CAL), and adherence to the Mediterranean diet. Chile harbors are one of the five Mediterranean-type ecosystems globally, with its Mediterranean climate present in the central region. This climate is comparable to other Mediterranean ecosystems found between latitudes 30° and 45° north or south, with west-facing coastlines, such as those in the Mediterranean Basin, California, southern Australia, and South Africa. Moreover, agricultural production in central Chile includes products like those in Mediterranean countries, with olive oil and wine being notably prominent [37]. Therefore, considering that traditional Chilean cuisine features various dishes utilizing ingredients and preparation methods akin to those of the Mediterranean diet, it represents a feasible and culturally relevant alternative for promoting a healthy lifestyle. In order to design a diet focused on denture wearers, food hardness has influenced chewing behavior.

The influence of the hardness of foods on chewing behavior is an area widely studied in dentistry [38]. Park et al. [39] analyzed the number of chewing cycles and the duration of chewing in relation to the hardness of a set of rice products in a group of adult subjects (26.28 ± 2.78) years and another group of elderly (79.53 ± 3.48). They found that as hardness increased, the duration and number of chewing cycles also increased in the young group. Laird et al. [40] found similar results using cooked sweet potato, chicken, celery, and raw sweet potato. Tonni et al. [38] conclude that hard foods increase all physiological masticatory parameters and suggest that a hard diet can improve mastication efficiency. Food texture influences in bolus particle size and saliva flow. Saliva incorporation to the bolus contributes to digestion, due to the presence of enzymes in saliva, and moisture and lubrication making, swallowing easier [41].

Traditionally, texture profile analysis (TPA) is used in food industry to assess food texture. TPA consists of two compression cycles where the food sample is compressed until it reaches a pre-established deformation. The two cycles simulate the chewing action. Rheological properties such as hardness, cohesiveness, and chewiness are described by TPA. The first force peak achieved in the first cycle is used as a measure of sample hardness. TPA helps in characterization of foods and its influence in individuals with compromised oral function [39,42].

In this context, a diet with high nutritional value that also requires the patient to exercise chewing could have clinical implications. The present study aims to evaluate the hardness of foods, by means of TPA [43], in a diet adapted to Chilean gastronomy, based on the Mediterranean diet. These results will be used as reference for a future dietary guideline that considers nutritional value and chewing exercise.

## 2. Materials and Methods

### 2.1. Groups of Foods Based on Mediterranean Diet

The MedDiet is largely described in the literature, emphasizing a high consumption of extra virgin olive oil, vegetables, fruits, cereals, nuts and legumes, a moderate consumption of fish and other meats, dairy products and red wine, and a low consumption of eggs and sweets [44].

Seven groups of foods are defined: dairy products, animal protein, vegetable protein, fruits, vegetables, cereals and grains and foods high in lipids or others. These are compounded by common products found in the markets of Chile. Table 1 shows the foods belonging to each group. These are traditional products in the Mediterranean diet [44]. Chanco cheese is a typical Chilean cheese. Jack mackerel is a fish cheaper than others present in Chilean cuisine, such as salmon. Husked wheatberries (mote con huesillo) [45] is a traditional Chilean dish. PACAM cream is a cream soup that is part of the Complementary Feeding Program for the Elderly (Programa de Alimentación Complementaria del Adulto Mayor, PACAM). The cooking procedures are based on local customs, taking into account not to overboil the food, as this reduces its nutritional value [46,47].

### 2.2. TPA Analysis

**Figure 1:**
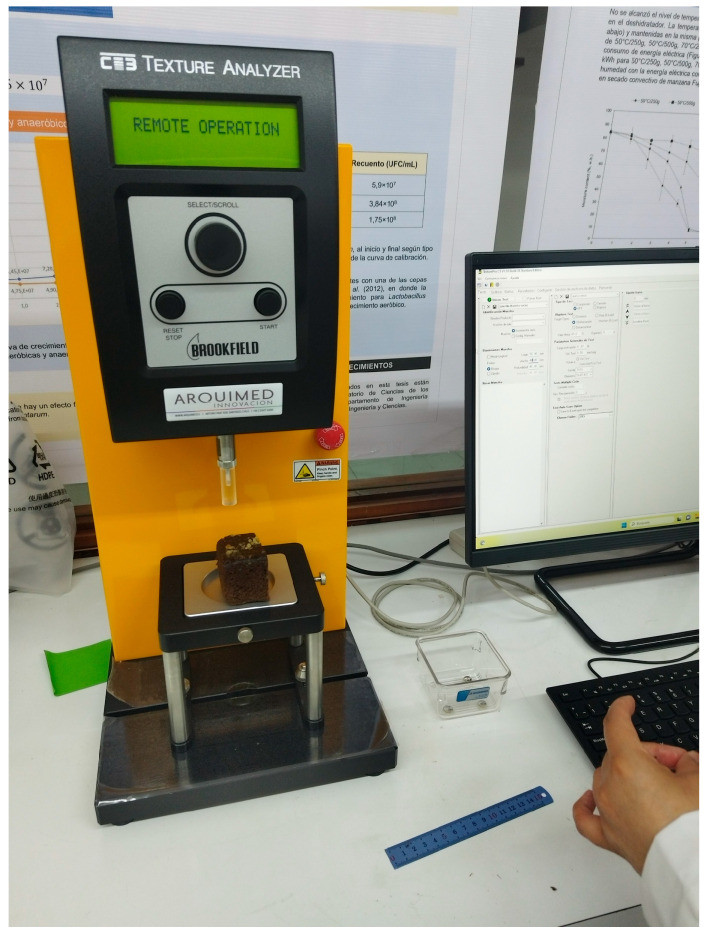
CT3 Texture Analyzer.

TPA analysis is a well-established method that provides data concerning the texture of the food tested. To make comparisons between foods that belong to the same group, each group was tested with particular conditions considering the texture of food. The analysis was performed using a CT3 Texture Analyzer (AMETEK Brookfield, Middleboro, MA, USA) and hardness was obtained (Figure 1). All foods were tested under the same conditions, subjected to two compression cycles, until 50% deformation was obtained. A speed of 0.5 mm/s, pre- and post-test speed of 2 mm/s, and 5 s rest after the first cycle were used during the test.

#### 2.2.1. TPA on Dairy Products

In this group, there are four types of cheese and yogurt. The products used were buttery cheese (Soprole, San Bernardo, Chile), chanco cheese (Quiyalles, Santiago de Chile, Chile), ricotta (Quiyalles, Santiago de Chile, Chile), soft unsalted cheese (Santa Ana de Huingán, Los Angeles, Chile), and light lactose-free yogurt (Colun, La Uniòn, Chile). These products were stored at 4 °C. Testing conditions are provided in Table 2.

Cheese-group test conditions were defined according to manufacturer recommendations and Rukiye et al. as the reference [48]. For ricotta and yogurt, manufacturer recommendations and guidance in Ismail et al. were used as the reference [49].

#### 2.2.2. TPA on Animal Protein

In this group, there are four types of meat and eggs. The products used were white eggs, canned jack mackerel (*Trachurus murphyi*), chicken thighs, turkey breast, and beef (sirloin tip). Testing and cooking conditions are provided in Table 3.

Meat samples were cut into 4 × 4 × 2 cm pieces. The egg sample was cut to achieve a 2 cm height in the thickest part. Aguirre et al. [50] was used as the reference.

#### 2.2.3. TPA on Fruits

There are five fruits in this group: kiwi (*Actinidia deliciosa*), orange (*Citrus aurantium*), banana (*Musa paradisiaca* L.), sweet cucumber (*Solanum muricatum*), and red apple (*Malus domestica* Borkh). Testing conditions are provided in Table 4.

Each product was cut to achieve a sample 2 cm in height. Banana and sweet cucumber were cut in the center of the fruit [51]. For fruit testing, a Magnes Tylor probe was used according to the manufacturer’s recommendation.

#### 2.2.4. TPA on Vegetables

There are 9 different products in the vegetables group: cooked beet (*Beta vulgaris*), tomato (*Solanum lycopersicum*), cooked broccoli (*Brassica oleracea* var. italica), lettuce (*Lactuca sativa* L.), cucumber (*Cucumis sativus* L.), cooked chard, (*Beta vulgaris* var. cicla), and raw carrot (*Daucus carota*). Testing and cooking conditions are provided in Table 5.

Leaf vegetable samples were prepared based on the work of Liu et al. [52]. Cucumber and carrots were cut in the middle of the food, as described in Nyorere et al. [53]. Beet was cut in 4 × 4 × 2 cm cubes. Tomato was cut 2 cm deep from the peel and with a height of 1 cm [54]. Probe selection was according to the manufacturer’s recommendation.

#### 2.2.5. TPA on Cereals and Grains

There were 8 foods in this group: oatmeal and milk, fusilli noodles, whole-wheat sliced bread, whole-wheat sliced bread with seeds, cooked quinoa, whole-grain rice, (Miraflores, Carozzi S.A., Santiago, Chile), couscous (Carozzi, Empresa Carozzi S.A., Santiago, Chile), and fresh, cooked husked wheatberries. Testing and cooking conditions are provided in Table 6.

Four oatmeal teaspoons were mixed with 200 mL of milk for one hour. Fusilli noodles were boiled for 10 min.

The oatmeal was tested using a beaker filled with 40 mL, reaching a height of 4 cm, using TA4-1000 probe (38.1 mm Ø) for liquid foods as ricotta and yogurt.

The bread was cut into pieces 2 cm in height and tested with a 36 mm diameter cylindric probe [55]. Fusilli noodles, cooked quinoa, whole-grain rice, couscous, and husked wheatberries were tested in the same way but, owing their nature, were contained in an Ottawa cell (447 mL) until reaching a height of 2 cm.

#### 2.2.6. TPA on High Lipid Food and Others

This group consisted of 6 foods: yogurt and flax, yogurt and chia, peach jam (Vivo, Empresa Carozzi S.A., Santiago, Chile), peanut butter (Manì King, Córdoba, Argentina), PACAM (Programa de Alimentación Complementaria del Adulto Mayor) cream, and avocado. For yogurt preparations, 600 mL of strawberry light without lactose yogurt (Cloun, La Uniòn, Chile), 12 g of flax, and 15 g of chia were used. PACAM cream was prepared according to package instructions. Testing and cooking conditions are provided in Table 7.

Semisolid samples were tested according to Ismail et al. [49] and manufacturer recommendations.

#### 2.2.7. TPA on Vegetal Protein

In the case of vegetal protein, this group comprised pulse foods: 4 mm and 5 mm diameter lentils (*Lens culinaris*), white beans (*Phaseolus vulgaris*), chickpeas (*Cicer arietinum*), and split peas (*Pisum sativum*). Testing and cooking conditions are provided in Table 8.

Legumes samples were tested according to Bragança et al. [56] and manufacturer recommendations.

### 2.3. Statistical Analysis

The present study is framed within the design of a balanced diet for patients. This is why the aim is to classify foods from different food groups. The statistical analyses conducted are aligned with this objective. The Shapiro–Wilk normality test was performed for each group. ANOVA and Tukey analyses were carried out to establish the presence of a significant difference within the groups and between each food when the samples showed a normal distribution. For non-normal samples, Kruskal–Wallis and Dwass–Steel–Critchlow–Fligner (DSCF) pairwise comparisons were used to analyze presence of a difference. Significant differences were judged at a level of *p* < 0.05. Jamovi 2.3.28 version was used. Comparisons between groups were not performed.

## 3. Results

All groups present a wide range of hardness. Kiwi, red apple, oatmeal and milk, and raw beet presented non-normal behavior (*p* > 0.05). All groups present significant differences (*p* < 0.05) according to ANOVA and Kruskal–Wallis (Table 9).

### 3.1. Dairy Group

**Figure 2:**
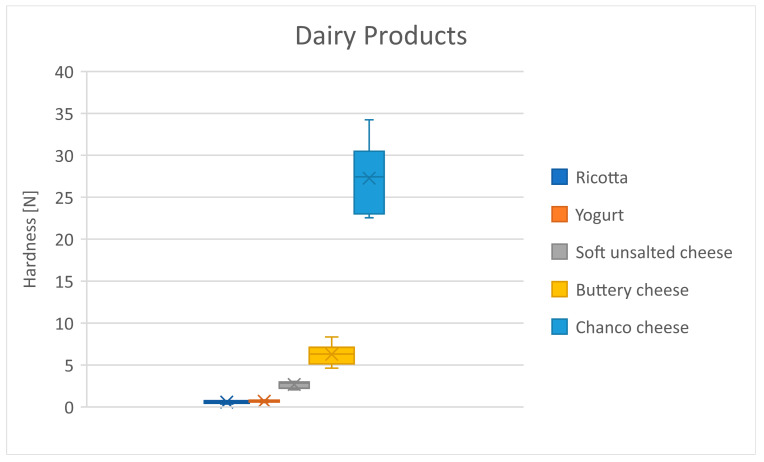
Distribution of hardness for the dairy products. Dots represent outliers. Crosses represent average.

Table 10 shows the mean hardness differences in the dairy group. Dairy products present a slowly growing trend (Figure 2), from ricotta to buttery cheese, to chanco cheese, where the hardness increases significantly. Yogurt and soft unsalted cheese are the softest foods, as expected since they are semi-solid (Table 10).

### 3.2. Animal Protein

**Figure 3:**
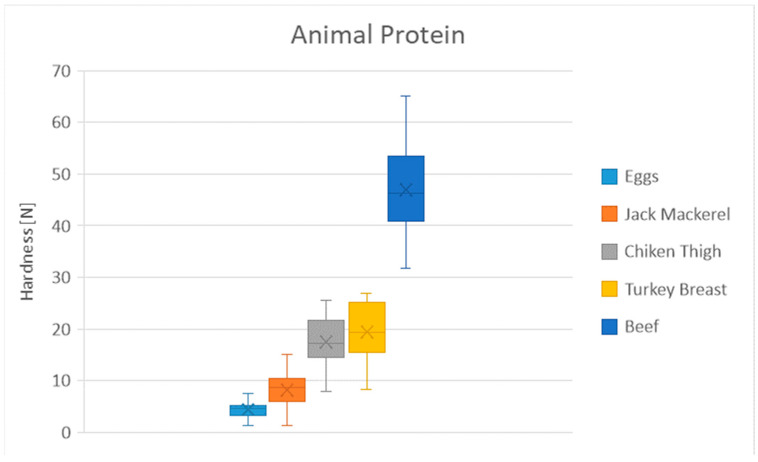
Distribution of hardness for the animal protein products. Dots represent outliers. Crosses represent average.

Table 11 shows the mean hardness differences in the animal protein group. A similar trend is observed until reaching beef, where the hardness increases significantly (Figure 3). Poultry meat, turkey breast, and chicken thigh have similar values. Eggs and jack mackerel cluster together at the lower hardness (Table 11).

### 3.3. Fruits

**Figure 4:**
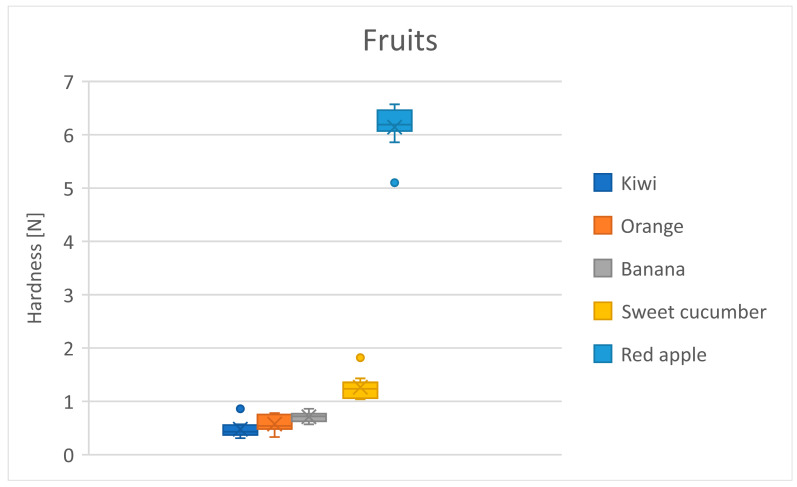
Distribution of hardness for the fruit products. Dots represent outliers. Crosses represent average.

Table 12 shows the mean hardness differences in the fruit group. Orange and kiwi are grouped as the least hard foods, which can be explained by the water content in these foods (Table 12). There is a trend of increasing hardness in the foods, with apple standing out in hardness compared to the others (Figure 4).

### 3.4. Vegetables

**Figure 5:**
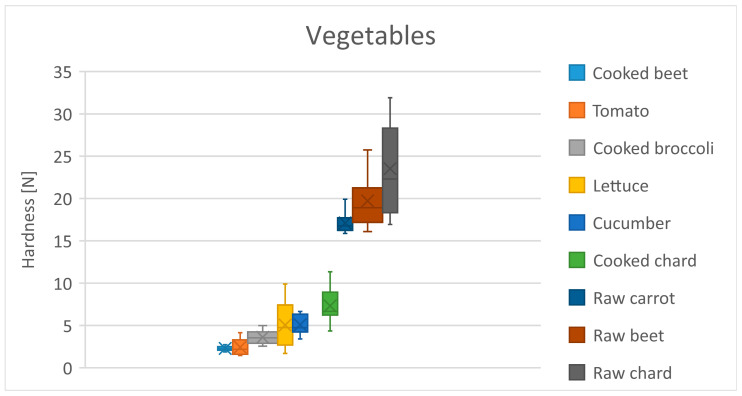
Distribution of hardness for the vegetables products. Dots represent outliers. Crosses represent average.

Table 13 shows the mean hardness differences in the vegetables group. In the vegetable group, raw vegetables exhibit greater hardness, not only compared to cooked ones, but also differ from the other vegetables by having a higher hardness (Table 13). Raw beetroot has hardness that is ten times greater than cooked beetroot. An increasing trend is observed in both the raw and cooked vegetable (Figure 5).

### 3.5. Cereals and Grains

**Figure 6:**
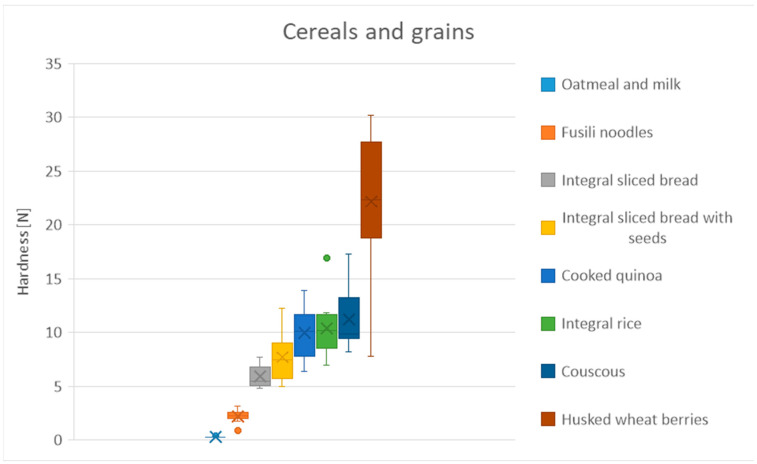
Distribution of hardness for cereals and grains products. Dots represent outliers. Crosses represent average.

Table 14 shows the mean hardness differences in the cereals and grains group. In this group, husked wheat berries are the hardest food, despite being the one that was cooked for the longest time (Figure 6). Couscous, cooked quinoa, and rice are grouped together and do not present significant differences despite the differences in cooking time. Oatmeal and milk is the softest food, which is consistent with the fact that it is the only semi-solid food in the group (Table 14).

### 3.6. High Lipid Foods and Others

**Figure 7:**
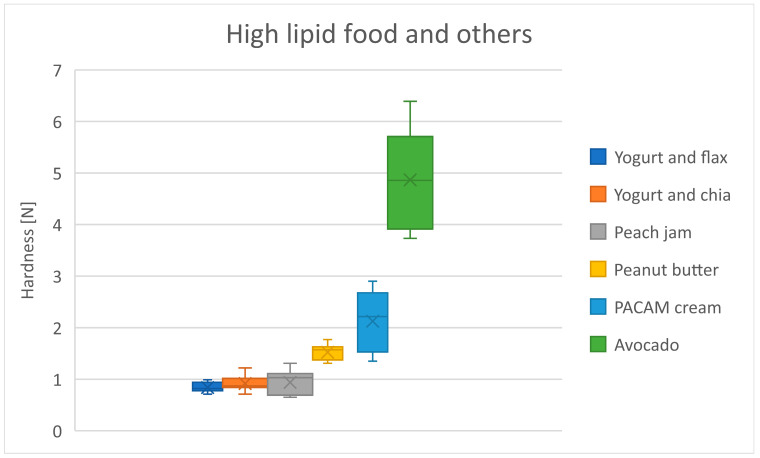
Distribution of hardness for high lipid food and other products. Dots represent outliers. Crosses represent average.

Table 15 shows the mean hardness differences in high lipid foods and others group. There is consistent hardness up to the peach jam, followed by a steady increase in hardness from peanut butter to avocado (Figure 7). Avocado is the hardest food, which is expected since it is the only solid food in the group. The hardness of the PACAM cream can be explained by the proportions used in its preparation (Table 14).

### 3.7. Vegetal Protein

**Figure 8:**
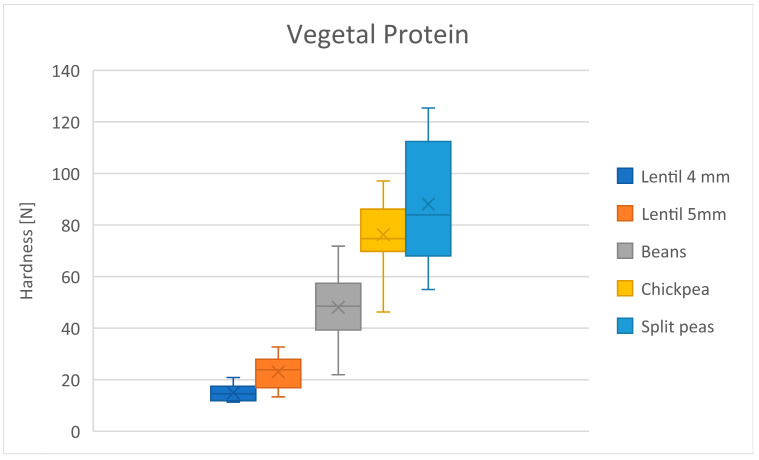
Distribution of hardness for vegetal protein products. Dots represent outliers. Crosses represent average.

Table 16 shows the mean hardness differences in the vegetal protein group. A constant rise in hardness is observed (Figure 8). Despite that lentil was cooked for 20 min and the other foods were cooked for 30 min, lentils are the softest food (Table 16).

## 4. Discussion

### 4.1. Food Hardness Ranking

The results from the dairy group can be explained by the protein content of the different cheese (Table 10). Młynek et al. [57] and Joshi et al. [58] found a positive correlation between the protein content and the hardness of the cheese. Chanco cheese, buttery cheese, and soft unsalted cheese present 24%, 22.5%, and 12% of the protein content, respectively. The results for animal protein (Table 11) are influenced by various factors. Masticability in meats is influenced by several key factors, including collagen content, meat texture, and preparation methods. Intramuscular collagen, particularly its cross-links, significantly affects meat tenderness; older cattle tend to have higher collagen levels, which correlates with increased toughness [59]. The initial texture of meat directly impacts masticatory variables, with tougher meats requiring more chewing and resulting in harder bolus characteristics [60]. In the fruit group, the results can be explained by the moisture content of each fruit. An inverse correlation has been observed between the moisture content and the fruit hardness measured by TPA [61,62]. According to the U.S. Department of Agriculture, moisture content in kiwi, orange, banana, and apple is 84%, 87%, 76%, and 85% respectively. In the cereals and grains group, cooked quinoa foods, integral rice, and couscous were among the hardest foods of its group (Table 14). This may be explained by their granulated form, which tends to compact under pressure. Quinoa exhibits superior compressive strength, attributed to its unique seed structure and higher protein content, which contributes to its resilience under stress and their starch composition and gelatinization behavior during cooking. For instance, quinoa exhibits a unique protein and fiber profile that contributes to its firmness and chewiness compared to rice and couscous, which are primarily starch-based and may become softer upon cooking [63]. For the vegetal protein group (Table 16), split pea was the hardest food and 4 mm lentil the softest. The results in this group may be explained by the compaction level of grains under the probe. The mechanical properties of cooked lentils and peas are influenced by several key factors, including cooking time, temperature, and the inherent composition of the legumes. Research indicates that prolonged cooking can lead to a decrease in firmness due to the breakdown of cell walls and starch gelatinization, which affects texture and overall mechanical strength [64]. The moisture content at the start of cooking is another critical factor, as it can alter the cooking dynamics and the resultant texture [65]. Different species exhibit varying mechanical properties post-cooking, influenced by their unique biochemical compositions [66].

### 4.2. TPA Analysis and Comparison of Results

Comparisons with results from other studies can be difficult, if not impossible, since TPA tests are not a standardized procedure. Furthermore, TPA test results depend on the conditions under which the test was developed. This is a well-known limitation of the TPA analysis [42,67,68,69]. Some researchers have addressed this problem by using the elastic modulus instead of the hardness value derived from TPA analysis [70,71,72]. Nevertheless, there are studies that have conducted texture analysis on various foods, making it possible to analyze the ranking of hardness obtained in other similar investigations.

In their study, Wee et al. [71] subjected 59 foods to TPA analysis. Some of these foods are like those analyzed here, making it possible to compare the relative hardness levels between them. The test conditions were 1 mm/s test speed, 30% strain, and a flat, circular compression plate of 75 mm diameter. Cooking conditions were not specified.

For animal protein, they found the following order in hardness level, from the highest to the lowest: chicken breast, beef, canned salmon, and boiled egg. In our study, the hardness order is beef, chicken breast, jack mackerel, and eggs. The difference in beef and turkey breast can be explained by differences in the cooking conditions. Nelum et al. [60] found that hardness in beef increases with cooking time and temperature. For cereals and grains, Wee found the following order: white rice, udon noodles, white sliced bread, and spaghetti noodles. In this study, the order was integral rice, integral sliced bread, and fusilli noodles. Finally, in vegetable group, Wee found the following order in hardness: raw carrot, tomato, and boiled broccoli. Our study found the following hardness order: raw carrot, boiled broccoli, and tomato. The differences observed in broccoli may be explained by different cooking conditions. This study has a higher speed and lower deformation than those in our study. A higher deformation speed is associated with a lower hardness record while a minor final deformation reports a minor hardness value [67]. No other study was found where several foods were tested by TPA analysis.

Comparisons between research are difficult since the results provided by TPA analysis are extensive variables, which means that the results depend on the mass, or more specifically, the thickness and the area of the sample [42]. Also, the deformation rate and degree of compression modify the results [68]. This may explain why so little research has been conducted on several foods instead of a specific food. On the other hand, not all foods can be treated in the same way and manipulated to obtain a homogeneous sample. Some foods can be cut into a specific form, like beets, potatoes, apples, some types of cheese, bread, etc. Others can be cut, but with an irregular surface, like eggs, meat, orange, and kiwi. Legumes and semi-solids like yogurt are cases where the sample must be contained to be tested.

Standardization of the TPA analysis requires not only fixing the speed and deformation level, but also the sample preparation. This should consider not only a standardized shape, but also which foods allow for such standardization. While cooking conditions influence the texture of the foods, they can be both standardized and adapted to local cuisine or the objectives of the study. An interdisciplinary analysis between materials science and food engineering can guide this process [42].

### 4.3. Dental Status and Food Selection

Characterization of food can help us understand certain behaviors of people with dental prostheses when selecting foods. In their study, Mercenes et al. [73] compared the perceived difficulty experienced by a group of dentulous patients and those with dentures. They found that patients with prosthesis have greater difficulty chewing foods. Apple was the food that presented the most difficulty for chewing, whereas orange showed no difference between groups. This is consistent with our results, as the apple was the hardest fruit found, while the orange was among the softest fruits. Regarding vegetables, they evaluated tomato, lettuce, and carrot. Carrot and lettuce presented similar difficulties, around 20%, for prosthesis users, while 40% of prosthesis users reported difficulty chewing carrot. This is related to the results of this study, where lettuce and tomato did not show significant differences in hardness, but raw carrot was among the hardest vegetables. Similar findings are reported by Watson et al. [74]. In this context, designing a diet focused on denture wearers is of interest to improve their nutritional condition [75].

These considerations have been taken into account in the present research, and each group was tested under specific conditions according to the relevant bibliography. Since there is no standard for groups or types of foods, adaptations were made to homogenize the test conditions. Additionally, no comparisons between groups were made because of this.

## 5. Conclusions

The results allowed for distinguishing different grades of hardness. Significant differences were found within each group and between foods. TPA analysis is the typical procedure to analyze food hardness, but several limitations are present in this method, which are well known among researchers. The use of TPA analysis for a diverse group of foods is a rarely explored field reported within the literature and needs to be standardized considering the nature of different foods. The design of standardized protocols could be future research that would fill the gap presented in the use of this test. Using these results together with food hardness as a control variable, it is possible to evaluate chewing in patients.

## Figures and Tables

**Table 1:** Detailed food groups based on the Mediterranean diet.

Dairy	Animal Protein	Fruits	Vegetables	Cereals and Grains	High Lipid Food and Others	Vegetal Protein
Ricotta	Eggs	Kiwi	Cooked beet	Oatmeal and milk	Yogurt and flax	Lentil 4 mm
Yogurt	Jack Mackerel	Orange	Tomato	Fusilli noodles	Yogurt and chia	Lentil 5 mm
Soft unsalted cheese	Chicken thigh	Banana	Cooked broccoli	Whole-wheat sliced bread	Peach jam	Beans
Buttery cheese	Turkey breast	Sweet cucumber	Lettuce	Whole-wheat bread with seeds	Peanut butter	Chickpeas
			Cucumber	Cooked quinoa	PACAM cream	
Chanco cheese	Beef	Red apple	Cooked chard	Whole-grain rice	Avocado	Split peas
			Raw carrot	Couscous		
			Raw beets	Husked wheatberries		
			Raw chard			

**Table 2:** Test conditions for the dairy products.

Foods	Cooking Process	Cooking Time (min)	Size Sample	Probe
Buttery cheese	Raw	-	4 × 4 × 2 cm	TA-10 (12.7 mm Ø)
Laminate cheese				
Soft unsalted cheese				
Chanco cheese				
Ricotta	Raw	-	Beaker filled with 40 mL, reaching a height of 4 cm	TA4-1000 probe (38.1 mm Ø)
Yogurt				

**Table 3:** Testing and cooking conditions for the animal protein products.

Foods	Cooking Process	Cooking Time (min)	Sample Size	Probe
Canned jack mackerel	Raw	-	4 × 4 × 2 cm height	TA-10 (12.7 mm Ø)
White eggs	Boiled	10		
Chicken thighs	Boiled	30		
Beef	Boiled	50		
Turkey breast	Baked 180 °C	80		

**Table 4:** Test conditions for the fruits.

Foods	Cooking Process	Cooking Time (min)	Size Sample	Probe
Kiwi	-	-	4 × 4 × 2 cm	Magnes Tylor (3 mm Ø)
Orange			2 cm	
Banana			2 cm	
Sweet cucumber			2 cm	
Red apple			4 × 4 × 2 cm	

**Table 5:** Testing and cooking condition for the vegetable products.

Foods	Cooking Process	Cooking Time (min)	Sample Size	Probe
Raw chard	Raw	-	5 × 5 × 1 cm	TA44 (4 mm Ø)
Cooked chard	Boiled	3		
Lettuce	Raw	-		
Cooked broccoli	Boiled	5	2 cm height	Magnes Tylor (3 mm Ø)
Tomato	Raw	-		
Cucumber	Raw	-		
Raw carrot	Raw	-		
Raw beets	Raw	-		
Cooked beet	Boiled	25		

**Table 6:** Testing and cooking conditions for cereals and grains.

Foods	Cooking Process	Cooking Time (min)	Sample Size	Probe
Oatmeal and milk	4 teaspoons with 200 mL of milk	60 resting	Beaker filled with 40 mL, reaching a height of 4 cm	TA4-1000 probe (38.1 mm Ø)
Fusilli noodles	Boiled	10	Ottawa cell (447 mL) filled until reaching a height of 2 cm	36 mm Ø
Whole-wheat sliced bread	Raw	-		
Whole-wheat sliced bread with seeds	Raw	-		
Cooked quinoa	Boiled	5		
Whole-grain rice	Boiled	10		
Couscous	Boiled	5		
Husked wheatberries	Boiled	30		

**Table 7:** Testing and cooking conditions for the high lipid food and others.

Foods	Cooking Process	Cooking Time (min)	Sample Size	Probe
Yogurt and flax	12 g of flax mixed with 600 mL of yogurt	-	Beaker filled with 40 mL, reaching a height of 4 cm	TA4-1000 probe (38.1 mm Ø)
Yogurt and chia	15 g of chi mixed with 600 mL of yogurt			
Peach jam	Raw			
Peanut butter	Raw			
PACAM cream	Package instructions			
Avocado	Raw		2 cm height	TA-10 (12.7 mm Ø)

**Table 8:** Testing and cooking conditions for the vegetal protein products.

Foods	Cooking Process	Cooking Time (min)	Sample Size	Probe
4 mm Lentil	Boiled	20	Ottawa cell (447 mL) filled until reaching a height of 2 cm	TA4-1000 probe (38.1 mm Ø)
5 mm Lentil				
White beans		30		
Chickpeas				
Split Peas				

**Table 9:** Mean and standard deviation for each food.

Group	Food	Mean [N]	±SD [N]
Dairy	Ricotta	0.6	0.2
	Yogurt	0.7	0.1
	Soft unsalted cheese	2.7	0.4
	Buttery cheese	6.3	1.2
	Chanco cheese	27.3	4.0
Animal Protein	Eggs	4.4	1.7
	Jack mackerel	8.2	3.7
	Chicken thigh	17.5	5.1
	Turkey breast	19.4	5.7
	Beef	47.0	9.3
Fruits	Kiwi *	0.5	0.2
	Orange	0.6	0.2
	Banana	0.7	0.1
	Sweet cucumber	1.3	0.2
	Red apple *	6.1	0.4
Vegetables	Cooked beet	2.3	0.3
	Tomato	2.4	1.0
	Cooked broccoli	3.6	0.8
	Lettuce	5.0	2.8
	Cucumber	5.1	1.1
	Cooked chard	7.3	2.1
	Raw carrot	17.1	1.2
	Raw beets *	19.7	3.3
	Raw chard	23.5	5.6
Cereals and Grains	Oatmeal and milk *	0.3	0.1
	Fusilli noodles	2.2	0.6
	Integral sliced bread	5.9	1.1
	Integral sliced bread with seeds	7.7	2.2
	Cooked quinoa	10.0	2.2
	Integral rice	10.4	2.8
	Couscous	11.2	2.7
	Husked wheat berries	22.2	6.7
High lipid food and others	Yogurt and flax	0.8	0.1
	Yogurt and chia	0.9	0.1
	Peach jam	0.9	0.2
	Peanut butter	1.5	0.2
	PACAM cream	2.1	0.6
	Avocado	4.9	0.9
Vegetal protein	Lentil 4 mm	14.9	3.3
	Lentil 5 mm	23.0	6.2
	Beans	48.1	14.3
	Chickpea	76.3	14.4
	Split peas	88.1	24.5

* Indicates non-normality results for the Shapiro–Wilk test (*p* > 0.05).

**Table 10:** Mean hardness differences for the dairy products. Results are expressed in newtons.

Dairy	Yogurt	Soft Unsalted Cheese	Buttery Cheese	Chanco Cheese
Ricotta	0.1	2.1	5.7 ***	26.7 ***
Yogurt	-	2.0	5.6 ***	26.6 ***
Soft unsalted cheese	-	-	3.6 ***	24.6 ***
Buttery cheese	-	-	-	21.0 ***

*** *p* < 0.001.

**Table 11:** Mean hardness differences for the animal protein. Results are expressed in newtons.

Animal Protein	Jack Mackerel	Chicken Thigh	Turkey Breast	Beef
Eggs	3.8	13.1 ***	15.1 ***	42.6 ***
Jack mackerel	-	9.3 **	11.2 ***	38.8 ***
Chicken thigh	-	-	1.9	29.5 ***
Turkey breast	-	-	-	27.5 ***

** *p* < 0.01, *** *p* < 0.001.

**Table 12:** Mean hardness differences of the fruits. Results are expressed in newtons.

Fruits	Orange	Banana	Sweet Cucumber	Red Apple
Kiwi	0.1	0.2 *	0.8 ***	5.7 ***
Orange	-	0.1	0.7 ***	5.6 ***
Banana	-	-	0.6 ***	5.4 ***
Sweet cucumber	-	-	-	4.9 ***

* p < 0.05, *** *p* < 0.001.

**Table 13:** Mean hardness differences for the vegetables. Results are expressed in newtons.

Vegetables	Tomato	Cooked Broccoli	Lettuce	Cucumber	Cooked Chard	Raw Carrot	Raw Beet	Raw Chard
Cooked beet	0.2	1.3	2.8	2.8	5.0 **	14.8 ***	17.4 *	21.3 ***
Tomato	-	1.2	2.6	2.7	4.9 **	14.7 ***	17.3 *	21.1 ***
Cooked broccoli	-	-	1.5	1.5	3.72 *	13.5 ***	16.1 *	20.0 ***
Lettuce	-	-	-	0.05	2.3	12.1 ***	14.7 *	18.5 ***
Cucumber	-	-	-	-	2.2	12.0 ***	14.6 *	18.5 ***
Cooked chard	-	-	-	-	-	9.8 ***	12.4 *	16.2 ***
Raw carrot	-	-	-	-	-	-	2.6	6.4 ***
Raw beet	-	-	-	-	-	-	-	3.8

*p < 0.05, **p < 0.01, ***p < 0.001.

**Table 14:** Mean hardness differences for the cereals and grains. Results are expressed in newtons.

Cereals and Grains	Fusilli Noodles	Integral Sliced Bread	Integral Sliced Bread with Seeds	Cooked Quinoa	Integral Rice	Couscous	Husked Wheat Berries
Oatmeal and milk	1.9 **	5.6 **	7.4 **	9.7 **	10.1 **	11.0 **	21.9 **
Fusilli noodles	-	3.7	5.5 **	7.8 ***	8.2 ***	9.0 ***	20.0 ***
Integral sliced bread	-	-	1.8	4.1	4.5 *	5.3 **	16.3 ***
Integral sliced bread with seeds	-	-	-	2.3	2.7	3.5	14.4 ***
Cooked quinoa	-	-	-	-	0.4	1.2	12.2 ***
Integral rice	-	-	-	-	-	0.8	11.8 ***
Couscous	-	-	-	-	-	-	11.0 ***

* p < 0.05, ** *p* < 0.01, *** *p* < 0.001.

**Table 15:** Mean hardness differences for high lipid food and others. Results are expressed in newtons.

High Lipid Foods and Others	Yogurt and Chia	Peach Jam	Peanut Butter	PACAM Cream	Avocado
Yogurt and flax	0.1	0.1	0.7 *	1.3 ***	4.0 ***
Yogurt and chia	-	0.0	0.6	1.2 ***	4.0 ***
Peach jam	-	-	0.6	1.2 ***	4.0 ***
Peanut butter	-	-	-	0.6	3.3 ***
PACAM cream	-	-	-	-	2.7 ***

* p < 0.05, *** *p* < 0.001.

**Table 16:** Mean hardness differences for the vegetal protein. Results are expressed in newtons.

Vegetal Protein	Lentil 5 mm	Beans	Chickpea	Split Peas
Lentil 4 mm	8.1	33.1 ***	61.3 ***	73.2 ***
Lentil 5 mm	-	25.0 **	53.2 ***	65.1 ***
Beans	-	-	28.2 ***	40.0 ***
Chickpea	-	-	-	12.0

** *p* < 0.01, *** *p* < 0.001.

## Data Availability

The data presented in this study are available on request from the corresponding author. The data are not publicly available due to no public database is available.
